# PhageTailFinder: A tool for phage tail module detection and annotation

**DOI:** 10.3389/fgene.2023.947466

**Published:** 2023-01-23

**Authors:** Fengxia Zhou, Han Yang, Yu Si, Rui Gan, Ling Yu, Chuangeng Chen, Chunyan Ren, Jiqiu Wu, Fan Zhang

**Affiliations:** ^1^ HIT Center for Life Sciences, School of Life Science and Technology, Harbin Institute of Technology, Harbin, China; ^2^ Department of Hematology, Department of Oncology, Boston Children’s Hospital, Harvard Medical School, Boston, MA, United States; ^3^ Department of Genetics, University Medical Center Groningen, University of Groningen, Groningen, Netherlands; ^4^ Anhui Province Key Laboratory of Medical Physics and Technology, Institute of Health and Medical Technology, Hefei Institutes of Physical Science, Chinese Academy of Sciences, Hefei, China

**Keywords:** phage, tail gene cluster, two-state HMM, DBSCAN, phage therapy

## Abstract

Decades of overconsumption of antimicrobials in the treatment and prevention of bacterial infections have resulted in the increasing emergence of drug-resistant bacteria, which poses a significant challenge to public health, driving the urgent need to find alternatives to conventional antibiotics. Bacteriophages are viruses infecting specific bacterial hosts, often destroying the infected bacterial hosts. Phages attach to and enter their potential hosts using their tail proteins, with the composition of the tail determining the range of potentially infected bacteria. To aid the exploitation of bacteriophages for therapeutic purposes, we developed the PhageTailFinder algorithm to predict tail-related proteins and identify the putative tail module in previously uncharacterized phages. The PhageTailFinder relies on a two-state hidden Markov model (HMM) to predict the probability of a given protein being tail-related. The process takes into account the natural modularity of phage tail-related proteins, rather than simply considering amino acid properties or secondary structures for each protein in isolation. The PhageTailFinder exhibited robust predictive power for phage tail proteins in novel phages due to this sequence-independent operation. The performance of the prediction model was evaluated in 13 extensively studied phages and a sample of 992 complete phages from the NCBI database. The algorithm achieved a high true-positive prediction rate (>80%) in over half (571) of the studied phages, and the ROC value was 0.877 using general models and 0.968 using corresponding morphologic models. It is notable that the median ROC value of 992 complete phages is more than 0.75 even for novel phages, indicating the high accuracy and specificity of the PhageTailFinder. When applied to a dataset containing 189,680 viral genomes derived from 11,810 bulk metagenomic human stool samples, the ROC value was 0.895. In addition, tail protein clusters could be identified for further studies by density-based spatial clustering of applications with the noise algorithm (DBSCAN). The developed PhageTailFinder tool can be accessed either as a web server (http://www.microbiome-bigdata.com/PHISDetector/index/tools/PhageTailFinder) or as a stand-alone program on a standard desktop computer (https://github.com/HIT-ImmunologyLab/PhageTailFinder).

## 1 Introduction

Bacteriophages are obligatory viral parasites of microorganisms such as bacteria, actinomycetes, spirochetes, and mycoplasmas (Gan et al., 2022). These viruses were first observed by Frederick Twort in England in 1915 (Twort, 1915) and were isolated and named by a French-Canadian microbiologist Felix D’Herelle in 1917 (D’Herelle, 2007). While bacteriophages target a narrow and specific population of bacteria, penicillin, discovered by Alexander Fleming in 1928, and other antibiotics affect a broader range of microbes (Salmond and Fineran, 2015). This wider spectrum and strong antibacterial activity of antibiotics resulted in the decrease of phage research, with only the former Soviet Union and some eastern European countries exploring the therapeutic utility of bacteriophages. However, the emergence of bacterial resistance, particularly during the last 2 decades, brought considerable challenges to the clinical treatment of infectious diseases. Managing multidrug-resistant bacterial infections in the future requires the development of new antibacterial drugs, finding new bacterial targets, and identifying ways of inactivating bacterial antibiotic-resistance genes. However, these approaches have high research and development costs and long research cycles, so they are unlikely to solve the growing problem of bacterial resistance in the short term. Thus, there is renewed interest in phage therapy (Zhou et al., 2022). Bacteriophages are often very specific, with some infecting only a single bacterial species, resulting in greater specificity and lower side effects than conventional antibiotics. In addition, phages can also be used for gene editing and surface display in bacteria, due to their rapid reproduction, high specificity, and easy transformation (Lin et al., 2017).

Based on morphologic features, bacteriophages can be divided into 13 families, and the most common of these is Caudovirales. Most of the phages are contained in 15 genera of three families (Bao et al., 2019). A typical bacteriophage usually has an icosahedral head, a hollow needle-like structure, and a tail. The latter typically consists of an outer sheath and a base that can be further subdivided into a tail wire and a tail needle (Maciejewska et al., 2018). Caudovirales are divided into Siphoviridae, Myoviridae, and Podoviridae, depending on whether their tails are long and non-shrinking, long and shrinking, or short (Dion et al., 2020). Phages are also classified depending on whether they lyse bacteria. While virulent phages (lysogenic phages) destroy their hosts, temperate phages (lysogenic phages) do not (Nobrega et al., 2018). The action of lysogenic phages follows a predetermined sequence. After the phage is adsorbed on the bacterial surface, enzymes in the tail structure penetrate the peptidoglycan layer of the host. This is followed by the penetration of the inner membrane, allowing the release of nucleic acid content into bacteria. The phage tail protein can also act to inhibit the phage nucleic acid being excreted. After the phage nucleic acid integrates with the host nucleic acid content, it undergoes extensive replication. These *de novo* synthesized nucleic acid strands can be reassembled with the simultaneously produced phage shell proteins, resulting in a new progeny of infectious particles. Finally, due to the action of cytolytic enzymes and/or perforin, the infected bacteria are lysed, releasing progeny phages to infect additional surrounding hosts (Chevallereau et al., 2022). This self-propagating infectious cycle can be safely used to treat bacterial infections without harming the organism carrying the bacteria.

Structures necessary for a phage to bind to the bacterial surface during the adsorption phase are collectively referred to as receptor binding proteins (RBPs). They can hydrolyze bacterial surface structures to assist the injection of nucleic acid. A single phage particle can have multiple RBPs, affecting the specificity of adsorption and influencing the range of hosts that can be infected. Although most RBPs are either tail spines, tail fiber proteins, or substrates in the tail structure, these components show a high degree of diversity and exhibit unexpectedly low sequence conservation. These factors make predicting tail motifs and the role of a given sequence extremely challenging. Several computational tools have been developed to deal with the complex task of predicting phage tail proteins. To create iVIREONS, Seguritan et al. (2012) trained artificial neural networks using amino acid frequency and isoelectric points as features to classify the phage tail proteins. The more recently developed VIRALpro tool (Galiez et al., 2016) used a support vector machine (SVM) model, considering average amino acid composition and average secondary structure composition to predict the phage tail proteins. Subsequently, DeepCapTail (Abid and Zhang, 2018) proposes a deep neural network using k-mer frequency as features to predict capsid and tail phage proteins. More recently, Cantu et al. trained an artificial neural network, PhANNs (Cantu et al., 2020), using amino acid composition and instability index as features to predict the capsid and tail phage proteins. However, these tools are limited to the prediction of well-characterized proteins, and their performance is extremely poor when attempting to characterize proteins with no previously described homologous structures. In addition, some of the algorithms run rather slowly, as they also take into consideration secondary structures and other features. Furthermore, as genes with related functions tend to cluster together in the viral genome, the algorithms generally only predict whether the protein is part of the tail, while ignoring the modularity of the larger structure.

Here, we describe the development of a novel tool, the PhageTailFinder, to predict phage-related proteins using a two-state hidden Markov model (HMM). This approach is based on a probabilistic algorithm (Mor et al., 2021), detecting putative phage modules by density-based spatial clustering of applications with the noise algorithm (DBSCAN) (Ester et al., 1996). The developed PhageTailFinder tool can be run either as a web server (http://www.microbiome-bigdata.com/PHISDetector/index/tools/PhageTailFinder) or as a stand-alone version on a standard desktop computer (https://github.com/HIT-ImmunologyLab/PhageTailFinder).

## 2 Materials and methods

### 2.1 Creation of custom phage tail-related protein databases

#### 2.1.1 Training and test sets

Phages were collected from the Millard Laboratory database (Chibani et al., 2019). Only the entries indicating “complete genome” in the DEFINITION field were included. The final number of phage genomes in the training set was 6,287 (Supplementary Table S1) and included 1,763 Myoviridae, 3,461 Siphoviridae, and 1,063 Podoviridae. Additional 992 complete genome sequences covering the three possible tail types were downloaded from the NBCI nucleotide database (http://www.ncbi.nlm.nih.gov/nuccore/) in November 2020 (Supplementary Table S2) as a test set to evaluate the performance of the model. Details of the taxonomic distribution of the phages in the training and test datasets can be found in Supplementary Figure S1.

#### 2.1.2 Tail and non-tail profiles

First, we defined keywords that could be used for identifying tail-related proteins. Bacteriophages with well-defined tail structures reported in the scientific literature were manually curated (Table 1). By analyzing the occurrence and frequency of keywords used in the NCBI annotations and counting the functional domains predicted by RPS-BLAST identified 10 keywords describing tail proteins. These were “tail,” “tube,” “sheath,” “fibre,” “spike,” “baseplate,” “needle,” “tape,” “Terms,” and “TermL.” Next, we used these keywords to search the entire training set to detect the tail state. These terms were also supplemented by functional domain annotation. The training set used to teach the algorithm to define the tail state consisted of 840 characterized domains (Supplementary Table S3). To define the non-tail state, domains without significant sequence similarity to tail sequences (Pfam domain similarities with E-value <1e-4) were selected. The final training set consisted of 3,412 characterized non-tail domains (Supplementary Table S4).

**TABLE 1:** 13 well-defined phage genomes used in the validation process.

Phage	Phage_Genome_ID	Phage_Species
*Bacillus* virus phi29	EU771092.1	Podoviridae
*Salmonella* virus P22	BK000583.1	Podoviridae
*Enterobacteria* phage T3	NC_003298.1	Podoviridae
*Enterobacteri*a phage T5	NC_005859.1	Siphoviridae
*Bacteriophage* SPP1	NC_004166.2	Siphoviridae
*Enterobacteria* phage lambda	NC_001416.1	Siphoviridae
*Lactobacillus* phage LL-H	EF455602.1	Siphoviridae
*Salmonella* phage SSU5	NC_018843.1	Siphoviridae
*Escherichia* phage T2	MH751506.1	Myoviridae
*Escherichia* virus T4	NC_000866.4	Myoviridae
*Escherichia* phage Mu	AF083977.1	Myoviridae
*Listeria* phage A511	DQ003638.2	Herelleviridae
*Salmonella* phage Det7	NC_027119.1	Ackermannviridae

### 2.2 General phage tail-related protein prediction workflow

#### 2.2.1 Tail-related protein annotation

The protein annotation algorithm for the detection of tail regions is a two-state HMM, where one hidden state corresponds to tail protein clusters (tail state), while a second hidden state represents the rest of the genome (non-tail state). To construct this two-state HMM, we converted all training set phage genomes into protein sequences and represented these as contiguous protein family (Pfam) domains. These were used to train the initial probability, transition probability matrix, and emission probability matrix of the HMM. Initial probability was derived by counting the number of the two domains in the training set. This indicated 0.2039 tail state and 0.7961 non-tail state probabilities. The transition probability represents the likelihood that the state of the next domain would be tail or non-tail, once the state of a current domain is known. In the training set, the transfer probability from tail state to tail state was 0.1712, from tail state to non-tail state was 0.8288, from non-tail state to tail state was 0.0203, and from non-tail state to non-tail state was 0.9797. For each hidden state, their emission probability indicates the likelihood that they belong to a given Pfam. The domain structure of each protein was annotated by comparing with the previously established tail and non-tail HMM database using HMMscan. The domain with a smallest e-value was assigned if multiple domains were annotated to one protein. The emission probability matrix was generated by counting the frequency of each Pfam in the tail and non-tail latent states in the training set. In addition to this comprehensive model trained using all phages, we separately trained corresponding models for the three morphologic classes of phages.

#### 2.2.2 Tail-related protein module detection

The tail module of a phage consists of a cluster of tail-related proteins. In this study, we used the DBSCAN algorithm to cluster predicted tail-related proteins. The distance between proteins was defined based on protein spacing instead of nucleotide distance spacing to eliminate the bias that could be caused by differences in protein length. DBSCAN is a clustering algorithm based on density space. The difference between this algorithm and K-means algorithm is that instead of using predetermined clusters, the algorithm infers the number of clusters based on data. The number of proteins in the phage tail module is generally indeterminate; therefore, the use of this algorithm is appropriate. DBSCAN relies on two key parameters, the value radius of the adjacent area around a certain point (eps) and the number of points at least contained in the adjacent area (minpts). Optimization of these parameters in DBSCAN was achieved by iteratively performing density clustering on tail proteins in the training set.

### 2.3 Evaluation criteria

The prediction performance of the PhageTailFinder was evaluated using the receiver operating characteristic (ROC) curve by plotting the false-positive rate (1—specificity) against the true-positive rate (sensitivity) based on the threshold change for phage tail protein prediction. The area under the ROC curve (AUC) is modeled independent of the prediction score threshold. Sensitivity (true-positive rate) and specificity (true-negative rate) are used as accuracy metrics to evaluate predictions. Moreover, precision is also used to evaluate the performance of the PhageTailFinder.

## 3 Results and discussion

### 3.1 Modularity of the phage tail

The phage tail is composed of a series of proteins that cooperate with each other. In well-studied phages, such proteins appear to be encoded adjacent to each other within the genome. To explore whether this was also true in less well-characterized examples, we conducted a cluster analysis of tail proteins. Although well-defined phages invariably contain only one tail cluster, there is still considerable uncertainty about the organization of the phage tail module throughout the 13 families of bacteriophages. Therefore, we used the DBSCAN algorithm to cluster potential tail components rather than pre-specifying the number of the clusters.

The radius of the adjacent area around a given point (eps) and the number of points contained in the adjacent area (minpts) are the two key parameters used by the DBSCAN algorithm. Combining these parameters, points can be divided into three categories: core points, border points, and outliers. We assigned points into these categories according to the following process: 1) a given point was selected arbitrarily (neither assigned to a cluster nor specified as an outlier), and its neighborhood (NBHD) (eps and minpts) was calculated to detect core points. If a point was determined to be a core point, it was used to build a cluster around it. Other points were set as outliers. 2) This process was repeated with neighboring points until a cluster was established. The directly density-reachable points were added to the cluster first, and then the density-reachable points. If points marked as peripheral are added, their state was reset to the edge point. Steps 1 and 2 were repeated until all points were classified as core points, edge points, or outliers.

**FIGURE 1:**
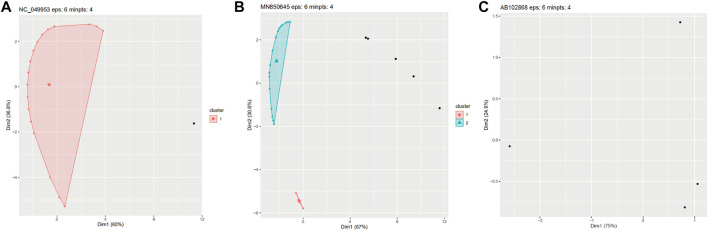
Three examples of clustering using the DBSCAN algorithm with the parameters: Eps = 6 and minpts = 4. **(A)** All tail proteins are clustered into one cluster. **(B)** Tail proteins are clustered into two clusters. **(C)** Proteins is too discrete to be clusters.

Through the iterative running of the algorithm until convergence, we established that setting the eps and minpts parameters at 6 and 4, respectively, resulted in the most reliable clustering, with the outcome mostly in line with the characteristics of tail protein distribution. Based on this clustering, most phages could be classified into three categories: 1) those where all or the vast majority of tail proteins formed a single cluster, with no or only few proteins being encoded elsewhere; 2) those where the tail proteins were clustered into two or three areas with a few discrete protein points; and 3) those where the number of proteins was too small or where the proteins were located too far apart to form a cluster (Figure 1).

A total of 961 phages were analyzed for tail modularity, including 642 Myoviridae, 293 Siphoviridae, and 26 Podoviridae family members. The results of this density clustering analysis are shown in Table 2. As indicated in the table, in 479 (74.6%) Myoviridae, 181 (61.7%) Siphoviridae, and 22 (84.6%) Podoviridae tail-related proteins were encoded in a single cluster. In contrast, 234 (36.4%) Myoviridae, 15 (5.1%) Siphoviridae, and only one (3.8%) Podoviridae phages had two-tail protein clusters. Phages containing three clusters were even less common, and four clusters were only detected in a small number of Myoviridae, with 25 (3.8%) phages organized in this manner. These results are in line with previous observations that tail proteins show strong clustering, with the majority of phages only containing one such cluster, demonstrating the feasibility of our approach to predict tail-related proteins based on the natural modularity. Nonetheless, more than one tail cluster was detected in some phages, a phenomenon potentially caused by horizontal transfer.

**TABLE 2:** Statistical results of cluster density analysis of 961 phages.

Morphology	Phage number	One cluster	Two clusters	Three clusters	Four clusters
Podoviridae	26	22 (84.6%)	1 (3.8%)	0	0
Siphoviridae	293	181 (61.7%)	15 (5.1%)	1 (0.37%)	0
Myoviridae	642	479 (74.6%)	234 (36.4^)	77 (11.9%)	25 (3.8%)

### 3.2 The PhageTailFinder algorithm detects tail-related proteins

HMM is a statistical model, named after the Russian mathematician Andrey Andreyevich Markov, used to describe a Markov process with hidden unknown parameters. The basis of HMM is the Markov chain. A Markov chain is a stochastic process in state space, where transitions occur from one state to another, and the probability distribution of the next state is determined by the current state. With the help of hidden state analysis, HMM estimates patterns in future observations. Since from the perspective of the PhageTailFinder tool, bacteriophage proteins are either tail proteins or non-tail proteins with natural modularity, the use of HMM is a promising potential approach for predicting whether a given protein is a tail component or not.

**FIGURE 2:**
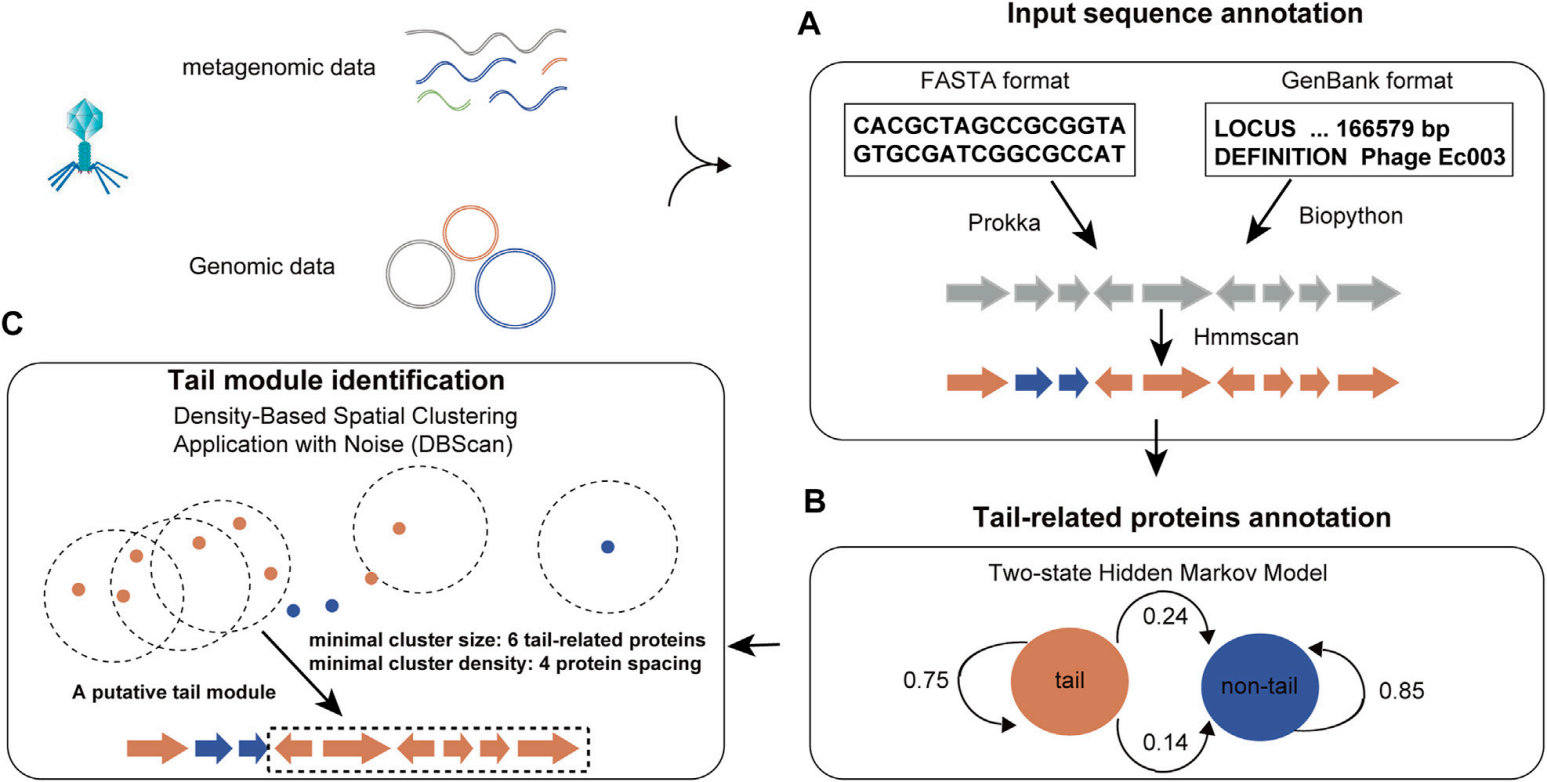
Flowchart of the PhageTailFinder. Flowchart illustrating the three-step tail module identification pipeline. **(A)** Annotating proteins in the phage genome and converting protein sequences into a string of Pfam domains. **(B)** Calculating posterior probabilities of the tail and non-tail hidden states to predict tail-related proteins. **(C)** Clustering the tail module using DBSCAN.

The challenge in optimizing this model lies in determining the implicit parameters of the process based on observable parameters. Proteins are functional units in biology, while domains are structural subunits necessary to maintain the structural integrity of a protein. Thus, domains belong to a level between secondary and tertiary structures in protein conformation, exhibit specific spatial conformation, and contribute to biological function indirectly. Typically, proteins consist of multiple domains, and protein–protein interactions occur between specific domains. It is important to note that while proteins with similar function may have widely different sequences, their domain level organization tends to show remarkable similarity. Such marked sequence differences in functionally related proteins pose considerable challenges in phage tail protein prediction. To overcome this issue, PhageTailFinder converts protein sequences into a string of contiguous Pfam domains by HMMScan (e-value < 1e-4). Probabilities are then calculated based on the domain frequency in the tail and non-tail training sets and the relationship between adjacent domains. The HMM for phage tail prediction was trained based on three important parameters: the transition probability matrix, emission probability matrix, and initial probability. This framework is illustrated in Figure 2. First, initial probabilities were constructed based on the frequency of tail and non-tail Pfam domains in the training set, resulting in a 0.2039 initial tail probability and 0.7961 initial non-tail probability. Next, the transition probability was calculated. These calculations indicated a probability of 0.0203 for a non-tail-to-tail transition and 0.9797 for a non-tail-to-non-tail transition. Finally, emission probabilities were determined based on the frequency of Pfam domains in the tail or non-tail hidden state. Since the PhageTailFinder solely relies on Pfam domain frequencies, it exhibits relatively little training bias and is capable of identifying new tail modules effectively.

The predictive power of the PhageTailFinder is primarily influenced by two parameters: the accuracy of HMM construction and the reliability of tail protein and the non-tail protein Pfam databases. The robustness of these key factors is heavily dependent on the number and representative nature of the phages included in the training set. To explore whether the domain feature was overfit due to the large number of phages in the training set, we tested the effect of reducing the size of the training set. While the initial training set contained 6,287 phages, this number was reduced to 2,000, 1,000, 500, and 100 in a stepwise fashion, randomly selecting 50 alternative training sets. It is important to note that as the number of phages present in the Myoviridae, Siphoviridae, and Podoviridae families is different. Therefore, the random training sets were selected to preserve the proportional representation of these phage families present in nature. Finally, we measured the performance of the models trained on these limited sets by calculating true-positive (TP) and false-positive (FP) rates (Supplementary Figure S2; Table 3). Somewhat surprisingly, as the number of tail-related Pfam present in the database decreased with the training sets getting smaller, the decrease in TP tail predictions was not particularly drastic. While the initial training set of 6,287 phages contained 840 tail Pfams, this was reduced by approximately 75% when the training set was limited to 100 phages. Yet, the corresponding TP rate only dropped by about 10%. This observation demonstrated the advantage of using Pfam as the observation feature since they can sufficiently represent tail domains even when the number of phages used in the training set was small.

**TABLE 3:** True-positive rate (TPR) of tail protein prediction models trained with a reducing number of phages.

Phage number	TPR = 1 (%)	TPR >0.8 (%)	TPR >0.6 (%)	Tail PRAM number
6287	35	58	84	840
2000	30	50	80	440–600
1000	33	57	81	539–595
500	30	55	83	416–480
100	20	42	70	215–265

### 3.3 Evaluation of the performance of the PhageTailFinder

To assess the reliability of PhageTailFinder predictions, we quantitatively evaluated the performance of the tool using a test set that consisted of 992 phage genomes and analyzing the rate of TP predictions, where real tail proteins were identified correctly, and FP rates correspond to actual non-tail proteins being classified as tail proteins. In this context, TP and FP indicate the accuracy and specificity of the algorithm. As shown in Supplementary Figure S3, the PhageTailFinder performed well in predicting the majority of phage proteins. Out of the 992 phages in the test set, the algorithm produced more than 80% accurate predictions in 570 phage genomes, accounting for more than half of the phages in the validated set. In addition, only about 10% of the phages had an FP rate of more than 10%, indicating the specificity achievable using the PhageTailFinder.

**FIGURE 3:**
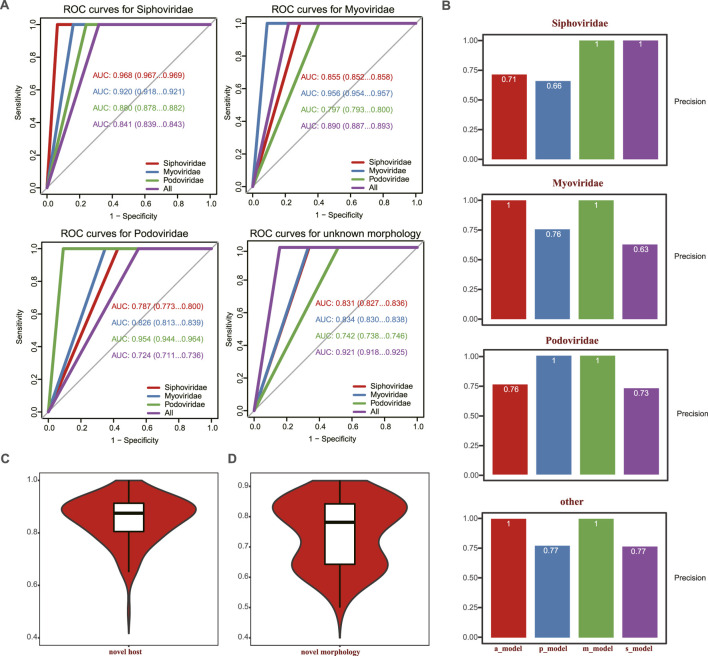
Comparison of the predictive power of the PhageTailFinder for 992 complete phages using four models. **(A)** ROC curve showing the predictive power of four models in the 992 complete phages, with the AUC values of 0.968, 0.956, 0.954, and 0.921. **(B)**. Precision values per morphology using corresponding models. **(C)** ROC curve showing the predictive power of a novel phage based on taxonomy. **(D)** ROC curve showing the predictive power of novel phage based on morphology.

To evaluate the performance of the model in identifying tail proteins in phages with specific morphological features, we subdivided the 992 phages in the test set into datasets containing only Myoviridae, Siphoviridae, or Podoviridae. Predictions were carried out in each morphology group, and we plotted the corresponding ROCs and calculated the AUC area and precision score. As shown in Figure 3, the best results were achieved when the predictions were made on phages within the same morphologic groups. Here, the AUC of predictions in Myoviridae, Siphoviridae, and Podoviridae reached 0.956, 0.968, and 0.954, respectively, the distribution of AUC per phage is illustrated in Supplementary Figure S4. When predictions were made across morphology groups, the performance of the model was higher when it was trained using the entire training set, containing all phage families. Under these circumstances, the AUC reached 0.8 (Figure 3A). The corresponding precision is shown in Figure 3B.

To evaluate the ability of the model to predict novel phage tail proteins, we created two additional dataset pairs. One pair consisted of 868 phage genera in the training dataset, referred to as previously “experienced” phages. In contrast, the other, “novel,” group consisted of 124 phage genera that were not present in the “experienced” dataset. The other dataset pair was divided based on morphologic features. It included 801 phages in the “experienced”—previously encountered—training set and 191 “novel” phages excluded from the training. By randomly sampling, “experienced” and “novel” phages of comparable sizes of 100 times, tail proteins were predicted in the “novel” subsets. The median values of novel tail AUC were 0.88 and 0.78, which could be achieved among previously “experienced” phages, where the prediction accuracy was 0.95 (Figures 3C, D; Supplementary Figure S4). Therefore, our method exhibits strong predictive ability for phage tail proteins, even in “novel” phages that have not previously appeared during model training.

### 3.4 Comparisons with other methods

We also conducted a comparison between the PhageTailFinder and other currently available protein analysis tools, comparing their precision and specificity in predicting phage tails in 13 extensively characterized phages. It is important to note that most published tools were not designed to discriminate between tail and non-tail proteins, so this could not be included in the comparison. Furthermore, while the VIRALpro, DeepCapTail, and PhANNs tools can identify tail proteins, these algorithms analyze phages at protein rather than the protein domain level. Therefore, we only compared the accuracy of phage protein annotation.

**FIGURE 4:**
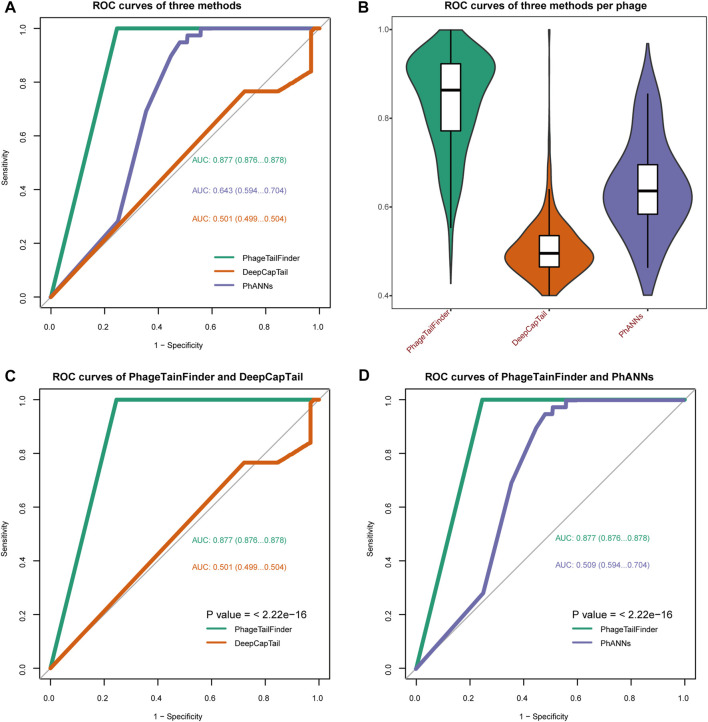
Comparisons of the performance of the PhageTailFinder with VIRALpro, DeepCapTail, and PhANNs. **(A)** ROC curve showing the predictive power of four tools when analyzing a test set consisting of 992 complete phage genomes. The resulting AUC values were 0.877, 0.643, and 0.501. **(B)**. Distribution of the AUC values per phage using four tools. **(C)** Bootstrap test on ROC between PhageTailFinder and DeepCapTail. **(D)** Bootstrap test on ROC between PhageTailFinder and PhANNs.

Phages with well-defined tail structures (phi29, SPP1, lambda, T3, T5, T7, T2, T4, LL-H, A511, Det7, SSU5, and P22) were used for validation purposes, and the TP and FP rates were used to assess algorithm performance. The TP rate achieved by the PhageTailFinder was consistently above 80%, PhANNs was 72%, DeepCapTail was 70%, while VIRALpro produced a TP rate below 50%. In addition, the FP rate achieved by the other algorithms was also high. Therefore, the PhageTailFinder showed higher precision and lower error rate in the identification of tail-related proteins. In addition, the average computing time of VIRALpro was over 2 min, while the PhageTailFinder did not exceed 1 min, a significant time advantage (Table 4). On the test dataset, the PhageTailFinder also showed significantly better performance, the AUC of PhageTailFinder achieves 0.877, while DeepCapTail and PhANNs are lower than 0.7 (Figures 4A, B), and the bootstrap test on ROC with *p*-value <2.22e-16 (Figures 4C, D).

**TABLE 4:** Comparison of the PhageTailFinder (PTF) with other prediction tools.

	PTF	VIRALpro	DeepCapTail	PhANNs
Last updated	2022	2016	2018	2020
Input type	FASTA/GenBank	FASTA	FASTA	FASTA
Timing	∼20s	>2 min	∼1 min	∼40s
Stand-alone	Yes	Yes	Yes	Yes
Tail protein prediction	Yes	Yes	Yes	Yes
Tail module prediction	Yes	No	No	No

### 3.5 Case study 1: Prediction of phage tail proteins for human gut virus

**FIGURE 5:**
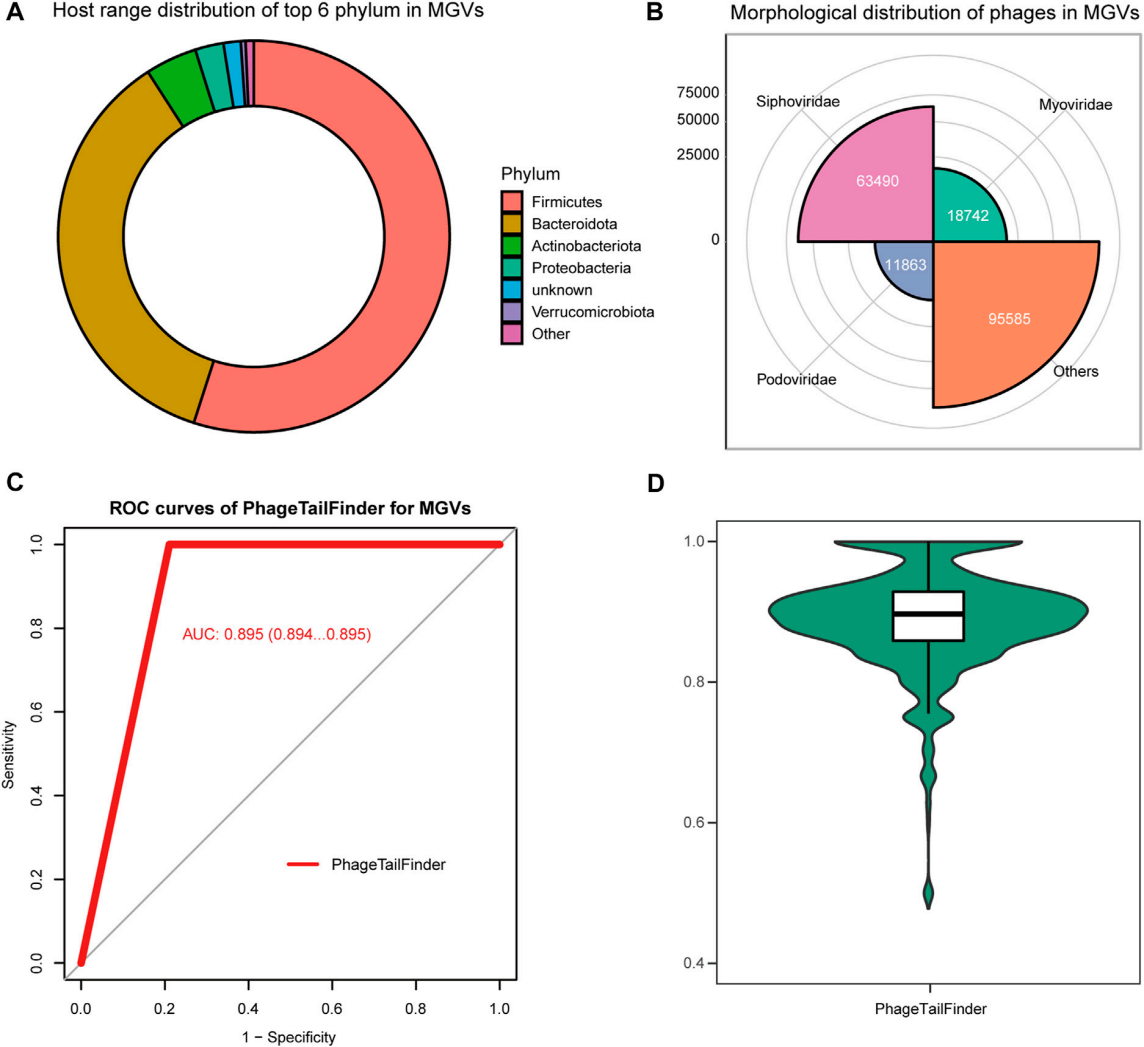
Performance of the PhageTailFinder in predicting phage protein in MGVs. **(A)** Taxonomy distribution of items detected as phage in MGVs. **(B)** Morphology distribution of items detected as phage in MGVs. **(C)** ROC curve showing the predictive power of models in the MGV set, with the AUC value of 0.895. **(D)** Distribution of the AUC values per phage using the PhageTailFinder.

The gut contains a complex microbial ecosystem with an important role in human health and development. Although often overlooked, phages are an abundant part of this microbiome (Reyes et al., 2010; Ogilvie et al., 2013) and may even be associated with the development of human diseases (Gogokhia et al., 2019). Bacteriophages represent the majority of viral particles in the gut (Ma et al., 2018). Despite their ubiquity, our understanding of viral genome diversity in the microbiome is limited. Stephen et al. performed large-scale viral genome characterization of bulk metagenomic data of human stool samples based on 61 previously published studies (Nayfach et al., 2021). The resulting metagenomic enterovirus (MGV) catalog contains 189,680 draft viral genomes, of which >50% appears to be complete, representing 54,118 candidate virus species. It is estimated that 92% of these MGVs are not represented in existing databases. These viruses are mainly distributed in *Firmicutes*, *Bacteroides*, and *Actinobacteriota*, and half of them are annotated as Caudoviricetes (Figures 5A, B).

Despite the annotation of potential host, bacterial species and predictions of host–virus relationships, the tail proteins, which are critical for designing phage therapeutics, have not been analyzed in detail. Thus, we attempted to identify the tail proteins in the cataloged 189,680 viral genomes using the PhageTailFinder. We used the tail and non-tail domains to annotate phage proteins using relatively conservative criteria (e-value < 1e-10) and subsequently used the PhageTailFinder to predict tail proteins based on the annotation results. We were able to identify 132,196 tail proteins, representing approximately 70% of viruses in the MVG catalog. The plotted ROC indicated an AUC area of 0.895 (Figures 5C, D). In summary, the PhageTailFinder could be successfully used to predict tail proteins from virally derived contigs in large datasets.

## 4 Conclusion

The vast majority of bacteriophages is currently uncultured and unclassified, and their specific hosts and infection strategies remain unknown. This population of organisms is often referred to as “viral dark matter” (Fitzgerald et al., 2021). Understanding the biology of these viruses is likely to bring major breakthroughs in medicine and basic sciences. Identifying phage tail module proteins is a key step in the process of understanding phage biology, as these proteins are essential during phage adsorption to the host. Recently, some computational tools have been devised to aid the prediction of the structural role of phage proteins. However, these methods exclusively rely on identifying sequence, structural, or physicochemical similarities to known phage proteins. Given the marked sequence variability of phage proteins and the relatively limited number of phages identified so far, the performance of such methods is greatly limited. In this study, we used the DBSCAN clustering algorithm to analyze known phage tail proteins. This work highlighted that phage tail proteins are modular. Based on this property, we proposed the PhageTailFinder, a novel tool that uses a two-state HMM to infer whether a protein in a phage is a tail or non-tail protein, independent of known sequence properties. We validated the performance of this algorithm on 13 extensively characterized phages and a selection of 992 phages collected from NCBI databases. In comparison, the PhageTailFinder outperformed previously devised algorithms in the accuracy and specificity of predicting phage tail proteins. We were also able to show that the PhageTailFinder had a better performance in identifying tail proteins not present in the training set. Finally, we annotated the tail proteins of 189,680 human enteroviruses, achieving correct tail annotation in 132,196 genomes (about 70%). Thus, the PhageTailFinder is a promising tool to support research in the potential therapeutic uses of phages. In addition, the novel algorithm is also significantly faster than the alternatives, making it suitable for high-throughput data analysis. We provide both a web server and a stand-alone version of the tool to users to allow flexibility in its use, according to the needs of the scientific community.

## Data Availability

The original contributions presented in the study are included in the article/Supplementary Material; further inquiries can be directed to the corresponding authors. The PhageTailFinder can be run either as a web server (http://www.microbiome-bigdata.com/PHISDetector/index/tools/PhageTailFinder) for general users to study individual inputs or as a stand-alone version (https://github.com/HIT-ImmunologyLab/PhageTailFinder) to process massive bacteria contigs from metagenomic studies.
