# Supplementary figures and images for: PhageTailFinder: A tool for phage tail module detection and annotation
Source: Front Genet. 2023 Jan 23;14:947466. doi: 10.3389/fgene.2023.947466 (PMC9901426; doi:10.3389/fgene.2023.947466)

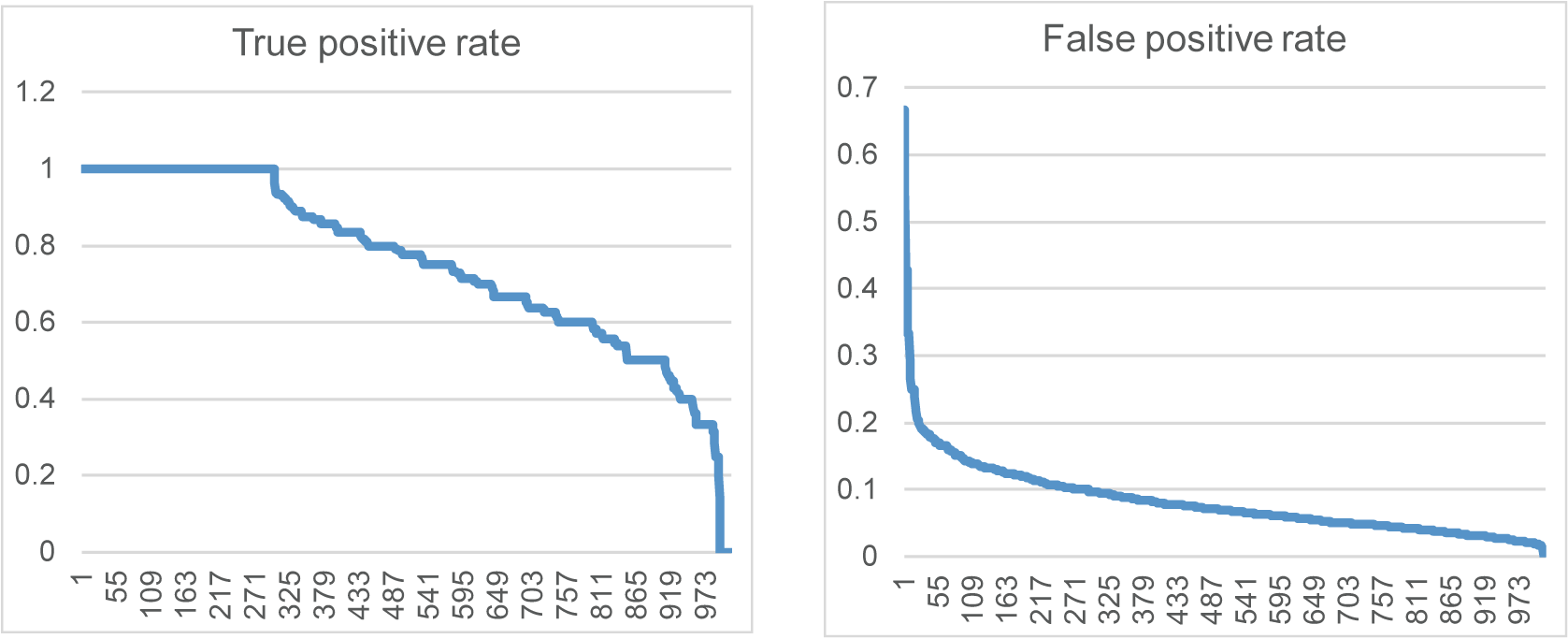

Supplement: Supplementary file 1 [file Image3.TIF]

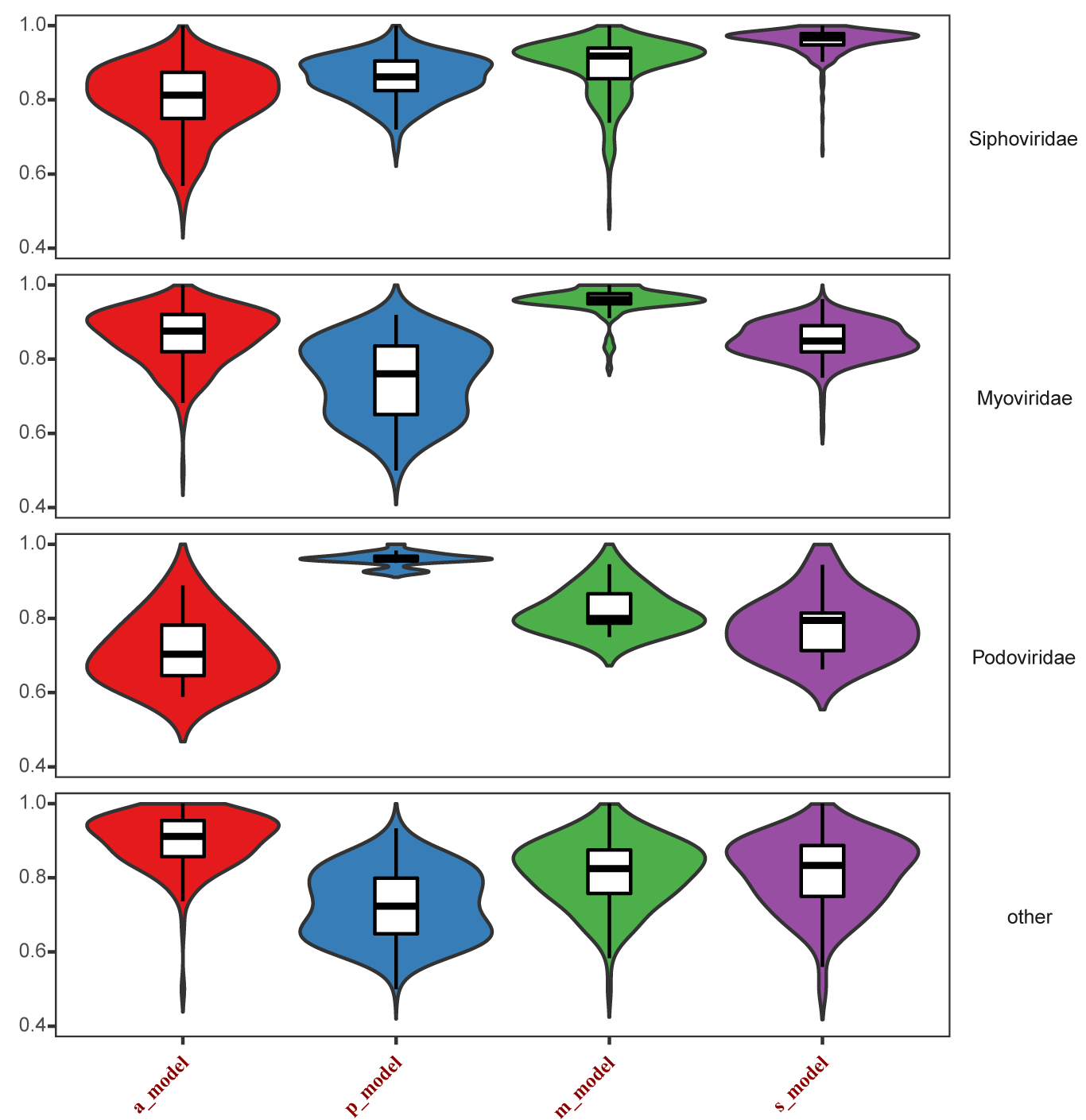

Supplement: Supplementary file 2 [file Image4.TIF]

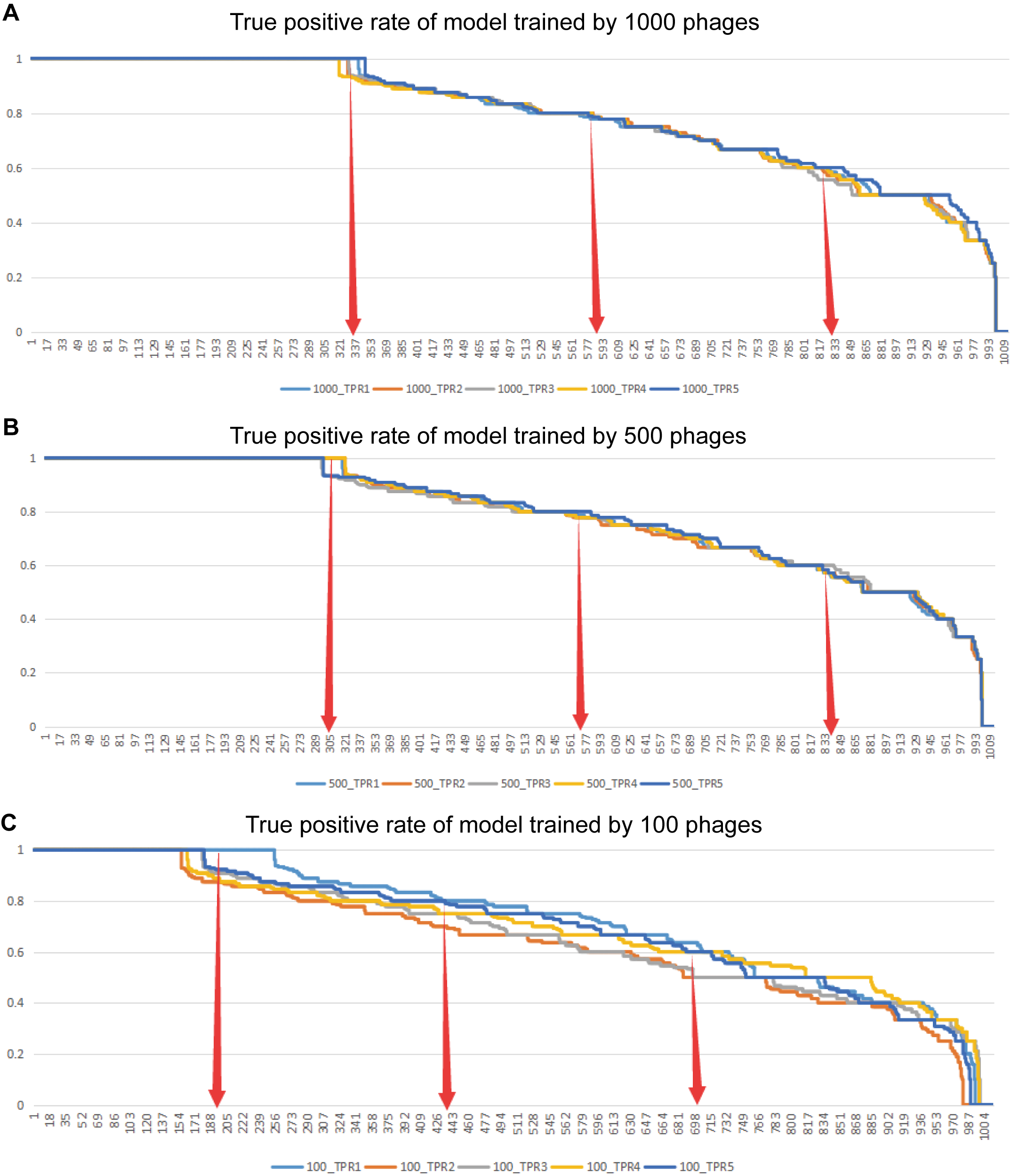

Supplement: Supplementary file 3 [file Image2.TIF]

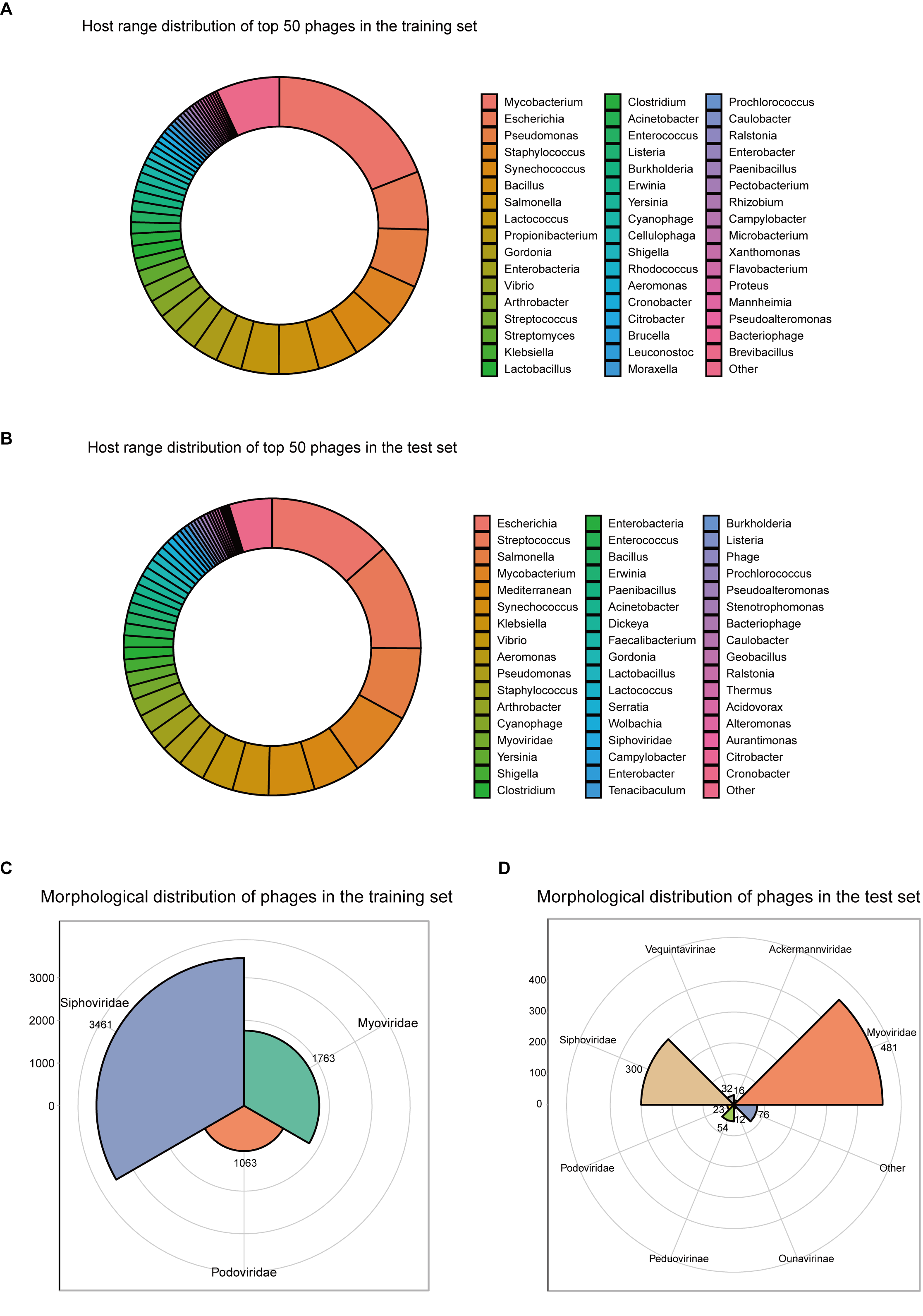

Supplement: Supplementary file 4 [file Image1.TIF]
